# Enterohepatic Migration of Fish Bone Resulting in Liver Abscess

**DOI:** 10.1155/2015/238342

**Published:** 2015-11-08

**Authors:** Chikwendu Ede, Sanju Sobnach, Delawir Kahn, Ahmed Bhyat

**Affiliations:** ^1^Department of Surgery, University of Witwatersrand, 7 York Road, Parktown, Johannesburg 2193, South Africa; ^2^Department of General Surgery, Groote Schuur Hospital and University of Cape Town, Anzio Road, Observatory, Cape Town 7925, South Africa; ^3^Department of General Surgery, Kimberley Hospital Complex, Du Toit Span Road, Kimberley 8300, South Africa

## Abstract

Liver abscess formation due to enterohepatic migration of a foreign body is extremely rare. Foreign body ingestion is generally an unconscious and painless event, thus complicating preoperative diagnosis in most patients. We report the case of a 61-year-old man who presented with secondary peritonitis from a ruptured hepatic abscess after an ingested fish bone migrated into the liver.

## 1. Introduction

Enterohepatic migration of an ingested foreign body with liver abscess formation is an extremely rare event. We report the successful management of a patient who presented with secondary peritonitis from a ruptured liver abscess, caused by an ingested fish bone that migrated into the liver.

## 2. Case Report

**Figure 1:**
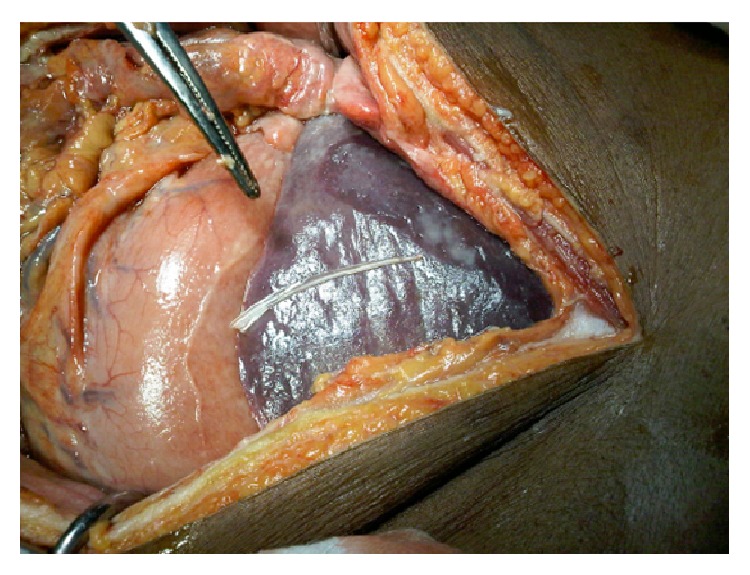
Fish bone extracted from abscess cavity.

A previously well 61-year-old man presented to our surgical unit with a three-week history of abdominal pain. Clinical examination revealed an anicteric pyrexial patient with generalized peritonitis. A chest roentgenogram showed no free air and a complete blood count and liver function tests were noted to be within normal limits. An abdominal ultrasound was highly suggestive of an abscess measuring 56 mm × 31 mm × 51 mm in the left liver lobe, but there was no radiologically demonstrable foreign body. An exploratory laparotomy was performed which showed free pus in the abdomen; the left lobe of the liver was adherent to the body of the stomach anteriorly. Gentle dissection revealed an abscess cavity in segment three (Couinaud) of the liver containing a fish bone measuring 6 cm in length (Figure 1).

Further inspection confirmed no fistulous communication between the stomach and the liver. The abscess was drained, fish bone was removed, and surgery was completed with peritoneal lavage and insertion of a corrugated drain. In the postoperative phase, the patient was managed with parenteral antibiotics and discharged uneventfully.

## 3. Discussion

The majority of ingested foreign bodies will pass through the gastrointestinal tract uneventfully. Although the incidence is less than 1%, sharp objects such as toothpicks, sewing needles, and fish bones can result in hollow viscous perforation [1]. An ingested foreign body may migrate to the liver and remain inert, only causing an abscess years later [2]. The first reported case of hepatic abscess due to gastrointestinal perforation by an ingested foreign body was reported by Lambert and colleagues in 1898, and since then only 59 cases have been reported in the literature [3, 4]. The usual site of perforation may not be obvious, but the stomach and duodenum are the most common culprit sites with impaction typically occurring in the left lobe of the liver [3]. In this case, it is likely that the fish bone migrated transgastrically into the left lobe of the liver, eventually forming an abscess. Symptoms are often nonspecific and the patient may not remember ingesting a foreign body. Preoperative diagnosis is difficult because of the rarity of the condition and the lack of a convincing history. A high index of suspicion is therefore invaluable. Plain roentgenogram will highlight radio opaque foreign bodies, while ultrasonography and computed tomography scans are useful in localizing the abscess and planning the surgical approach. Open surgery and abscess drainage remain the mainstay of the treatment for patients with foreign bodies lodged in the liver [3]. Minimally invasive techniques such as interventional radiology or laparoscopic surgery should be reserved for carefully selected cases, where both sepsis control and foreign body retrieval can be performed safely.
